# Cardiac Reoperation in a patient who previously underwent omentoplasty for postoperative mediastinitis: a case report

**DOI:** 10.1186/1749-8090-6-35

**Published:** 2011-03-24

**Authors:** Mehmet S Bilal, Onur Gürer, Ahmet Kırbaş, Yahya Yıldız, Ahmet Çelebi

**Affiliations:** 1 Department of Cardiovascular Surgery, Medicana Hospitals Camlica, Istanbul, Turkey; 2 Department of Anaesthesiology and Reanimation, Medicana Hospitals Camlica, Istanbul, Turkey; 3 Department of Pediatric Cardiology, Dr. Siyami Ersek Thoracic and Cardiovascular Surgery Center, Istanbul, Turkey

## Abstract

Sternal infection has become a rare but challenging problem with significant mortality and morbidity rates since the introduction of sternotomy. Reported rates of mediastinal and sternal infection range from 0.4% to 5%. The ideal reconstruction after sternal debridement is still controversial. Different methods, such as debridement and open packing with continuous antibiotic irrigation, or sternectomy with omental or muscle transposition have been proposed. In this study, we present the cardiac reoperation of a 52 year old man with corrected transposition of great arteries (c-TGA) who had undergone a previous omentoplasty for postoperative mediastinitis.

## Introduction

Sternal infection has been a challenging problem with high mortality and morbidity rates since the introduction of sternotomy in 1957 [1]. Mediastinitis after cardiac surgery is still an important complication associated with significant morbidity and mortality [2,3]. Mediastinal and sternal infection rates range from 0.4% to 5%.

As the subsequent septicemia and sepsis targeting the heart, the sutures lines and prosthetic conduits or valves can be life-threatening; a rapid and effective treatment is required to avoid high mortality in these patients. Optimal treatment for poststernotomy mediastinitis remains controversial.

In this study, we present the cardiac reoperation of a 52 year old man with corrected transposition of great arteries (c-TGA) who had undergone a previous omentoplasty for postoperative mediastinitis.

## Case Report

A 52 year old man was admitted to our clinic with shortness of breath and tachycardia. His past medical history included replacement of the mitral valve (biprosthesis 29 Sorin) and interposition of a valved conduit (25 mm Shelhigh) between the left ventricle and the pulmonary artery with a diagnosis of c-TGA, right atrioventricular valve (AV) insufficiency and pulmonary stenosis two years prior to presentation. His postoperative course was complicated by mediastinitis (blood cultures and exudate of the surgical wound were positive for methicillin-resistant Staphylococcus aureus), which required long-term antibiotic treatment and debridement of necrotic sternal fragments without success. Eventually, an omentoplasty (release of the greater omentum, sparing both vascular pedicles and short gastric vessels, with tunneling to the anterior mediastinum via upper midline laparotomy) was performed, sternum was closed with Robicsek type closure and the wound with a subcutaneous tissue and skin. The patient was discharged one month after the surgery. Upon presentation, his physical examination revealed a high grade systolic murmur at the right upper sternal border, decreased breath sounds and fine rales at lung bases, hepatomegaly and peripheral oedema. His blood pressure was 100/60 mmHg and his heart rate was 102 beats per minute. Cardiomegaly and bilateral pleural effusions were observed on chest x-ray. Echocardiographic examination revealed evidence of significant narrowing at the left ventricular-to-pulmonary artery (LV-PA) conduit (peak systolic instantaneous gradient of 130 mmHg), along with significant narrowing (a peak gradient of 29 mmHg and a mean gradient of 20 mmHg) and moderate regurgitation of the right AV bioprosthetic valve. The right atrium was dilated. Upon reviewing these findings, reoperation, in order to replace the prosthetic AV valve and the LV-PA conduit, was planned.

**Figure 1:**
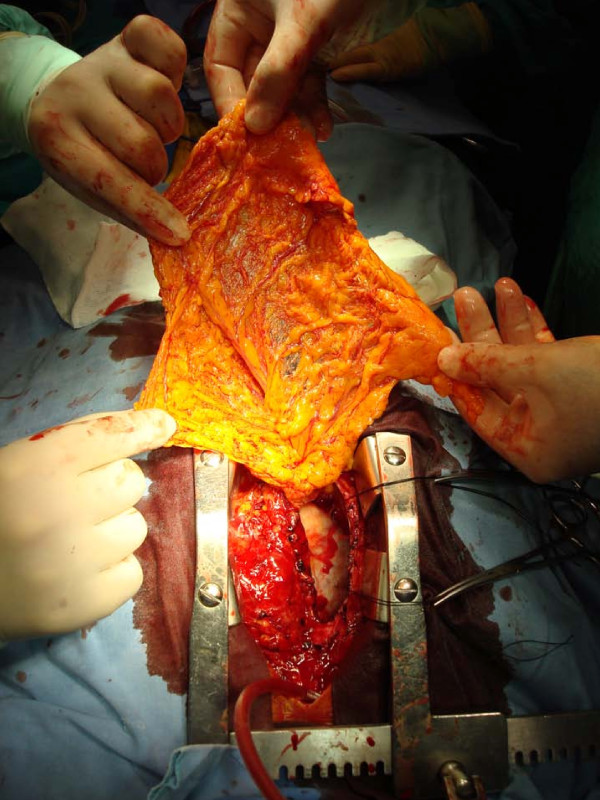
**The view of the preparation of the omentum**.

**Figure 2:**
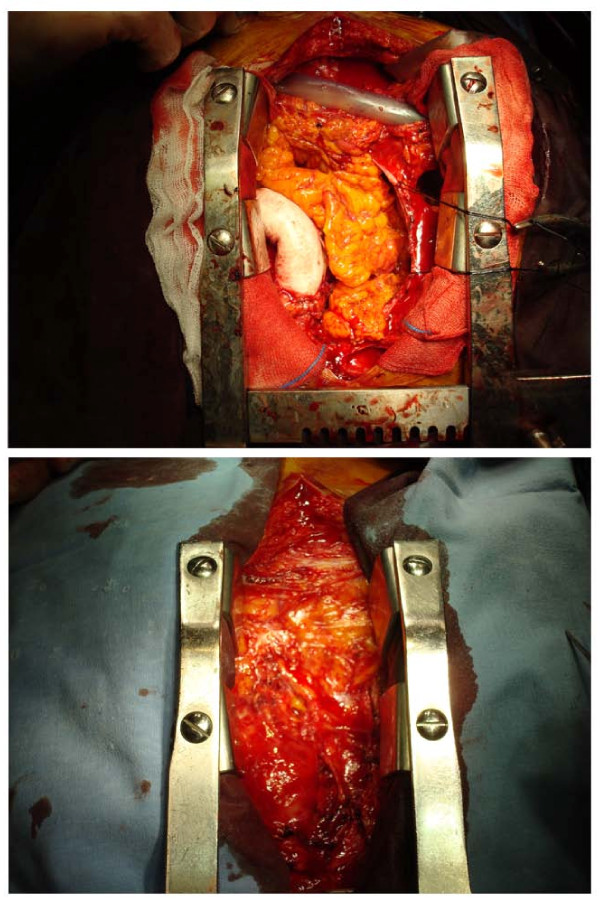
**The placement of omentum into the mediastinum and its reattachment**.

A median sternotomy was performed. Omentum was prepared carefully and protected with warm compresses (Figure 1). The right atrial pressure was 20 mmHg. Cardiopulmonary by-pass (CPB), utilizing femoral venous and arterial cannulation, was performed. Mitral bioprosthesis was replaced with a 29 mm St. Jude mechanical valve. On inspection, it was evident that the narrowing was at site of the previous ventriculotomy. No evidence of degeneration was observed at the valved conduit therefore the conduit was excised prior to the valve. After enlarging the original ventriculotomy, a 24 mm polytetrafluoroethylene (PTFE) tube graft was interposed between the LV and proximal conduit just below the valve. Normal sinus rhythm was reestablished, and CPB was discontinued without the need for inotropic support. Omentum was placed in the mediastinum and reattached (Figure 2). Sternum was closed with conventional sternal closure. Post-operative right atrial pressure was 10 mmHg. Wound healing was uneventful and the patient was discharged on post-operative Day 11. At the time of writing, he is at home, with satisfactory activity for his age and no signs of recurrent infection.

## Discussion

Postoperative sternal osteomyelitis is a rare but serious problem after cardiac surgery as the subsequent sepsis targeting the heart, suture lines, and prosthetic conduits or valves can be life-threatening [1,4,5]. Recent advances in cardiac surgery have enabled the surgical treatment of an increasing number of elderly and immuno-suppressed patients with multiple risk factors. However, despite efforts to control hospital infections and delivery of antibiotic treatment, the incidence of mediastinitis has remained constant over the years. Therefore, efforts to avoid high morbidity and mortality in these patients, has been required.

In 1963, antibiotic irrigation, debridement, and sternal re-closure were introduced [4]. After that, in 1976, Lee and colleagues [5] described complete excision of the sternum with wide debridement of costal cartilages, transposition of the omentum to the mediastinum with primary closure, while Jurkiewicz and colleagues [6] used muscle flaps. In 1995, Banic and colleagues [7] reported the use of free latissimus dorsi flap in cases of extensive sternectomy. In current practice, the most commonly utilized muscles for sternal reconstruction are the pectoralis major, rectus abdominus, latissimus dorsi and greater omentum.

Pairolero and Arnold [8] reported that, they primarily chose to obliterate the mediastinal space using omentum when previous interventions with different muscles have been unsuccessful. Omental flaps have several advantages. After complete or partial excision of sternum, the omental flap fills the mediastinal space and obliterates the dead space. The flap contains large number of immunologically active cells likely to be responsible for its anti-infective properties. As the omentum has extensive vascularization, and neovascularization potential, the increased blood supply leads to a higher concentration of antibiotics at the infection site. By absorbing wound secretions, the omental flap eliminates substrates for bacterial growth. Harvesting can be performed rapidly without the need for specialist knowledge, thus it can be undertaken by every surgeon [9].

The greatest disadvantage of utilizing the omentum in postoperative sternal osteomyelitis treatment is the need for a laparotomy. Laparotomy adds substantial surgical trauma in patients who are already very sick. On the contrary, the risk of potential peritoneal contamination seems to be negligible. Laparotomy may lead to postoperative pain that may interfere with the patient's ventilatory dynamic and may cause mucus retention, with possible respiratory infections. Furthermore, because of the postoperative ileus, it is more difficult to set the glucose values back to normal in diabetic patients [10].

Although omentoplasty is effective in mediastinitis treatment, it is a relative contraindication for future cardiac interventions through median sternotomy. The omental tissue has an excellent blood supply that limits the spread of infection. However, it also has adhesive properties that promote strong pericardial adherences and new vascular anastomosis with adjacent vessels that make a future sternotomy a real surgical challenge that no cardiac surgeon would like to face. Right or left thoracotomy may be a good alternative for these patients if coronary artery bypass grafting or valve surgery is to be performed, but not for other complex surgical procedures in which median sternotomy is mandatory [11].

## Conclusions

Omentoplasty for previous mediastinitis should not be considered a major contraindication for cardiac reoperations. Surgery is complex but technically possible. It is our belief that omentoplasty provides extra security in reoperations and safe to use in resternotomies.

## Competing interests

The authors declare that they have no competing interests.

## Authors' contributions

MSB drafted the manuscript. OG conceived the study and participated in its design and coordination. AK collected data about the patient. YY participated in the patient follow-up. AÇ participated in the study design and coordination. All authors read and approved the final manuscript.

## Consent statement

Written informed consent was obtained from the patient for publication of this case report and accompanying images. A copy of the written consent is available for review by the Editor-in-Chief of this journal.
